# Micromanagement in the gut: microenvironmental factors govern colon mucosal biofilm structure and functionality

**DOI:** 10.1038/npjbiofilms.2015.26

**Published:** 2015-12-16

**Authors:** Rosemarie De Weirdt, Tom Van de Wiele

**Affiliations:** 1 Laboratory of Microbial Ecology and Technology (LabMET), Ghent University, Gent, Belgium

## Abstract

The human gut microbiome provides us with functional features that we did not have to evolve ourselves and can be viewed as a structured microbial community that operates like a microbial organ within the human host. A minor but important part of this microbiome is the ability to colonise and thrive within the mucous layer that covers the colon epithelium. These mucosal microbes intimately interact with the intestinal tissue and seem to be important modulators of human health. Embedded in the host-secreted mucous matrix, they form a ‘mucosal biofilm’ with a distinct composition and functionality. In this review, we provide evidence that six specific (micro)environmental factors near the colon mucosa shape and determine mucosal biofilm formation and stability, that is, (1) mucous rigidity, (2) gradients of fluid shear, (3) radial oxygen gradients, (4) secretions of host defense molecules, (5) the presence of a rich but challenging nutrient platform and (6) the presence of niches at the colon epithelial surface. In addition, it appears that microbes actively participate in shaping their mucosal environment. Current insights into the interaction between mucosal microbes and their environment are rather limited, and many questions regarding the contribution of mucosal biofilm functionality and stability to human health remain to be answered. Yet, given the higher potency of mucosal microbes than their luminal counterparts to interact with the host, new insights can accelerate the development of novel disease-preventive or therapeutic strategies.

The human colon harbours a highly dense microbial community of 10^11^–10^12^ cells per gram of gut content.^1,2^ Although the majority of this community thrives in the lumen, few microbes can be found within the protective mucous layer that covers the epithelial cells (10^5^–10^6^ cells per ml of mucus).^3^ These microbes form the colon ‘mucosal biofilm’. Mucosal biofilms were previously defined as microbial biofilms that are unique to the mucosal environment.^4^ In contrast to biofilms that grow on inert surfaces, they are modulated by host inflammatory responses, and host proteins and cells contribute to the composition of the extracellular matrix.^4^ Healthy colon mucosal biofilms typically have low microbial densities,^5^ and growth of complex mushroom-shaped structures that are common for mature biofilms (indicated as stage 4 by Stoodley *et al.*^6^) have thus far not been observed. Hence, from a microbial point of view, we infer that the colon mucosal biofilm should be regarded as an immature biofilm.

The mucosal biofilm is unique in composition compared with the microbial populations that colonise the large intestinal lumen and make up faeces.^7–9^ At the phylum level, colon mucosal biopsies are enriched in Firmicutes (especially members of the *Lachnospiraeceae* and *Ruminococcaceae* families) compared with luminal or faecal samples.^10–12^ At the genus level, abundant and ubiquitous mucosal members are *Bacteroides*, *Faecalibacterium*, *Roseburia*, *Blautia* and a series of lactic acid bacteria classified as *Leuconostoc*, *Weissella*, *Lactococcus* and *Streptococcus*.^13,14^

Mucosal microbes intimately interact with the gut epithelium and gut-associated lymphoid tissue,^15–17^ and are increasingly evidenced to be important modulators of human health. On one hand, their abundance and prevalence is associated with disease. Low levels of *Faecalibacterium prausnitzii* have been correlated with active IBD and infectious colitis,^18^ quiescent ileal Crohn’s disease^19^ and colorectal adenomas.^20^ On the other hand, mucosal species (*Bacteroides fragilis*, *Lactobacillus reuteri*) have been shown to protect against experimentally induced colitis in animal models.^21,22^ Interestingly, members of the mucosal microbiome are found to possess distinct functionalities that are not expressed in standard *in vitro* set-ups. One such example is the extremely oxygen-sensitive *F. prausnitzii* species, which has been described to use an exogenous flavin-thiol electron shuttle to cope with elevated oxidative stress in the mucosal environment.^23^ The case of *F. prausnitzii* is one of many examples showing how physicochemical conditions in the gut mucosa determine microbial colonisation processes.

We review the current knowledge on how the specific physicochemical conditions near the colon mucosa shape the mucosal biofilm composition and functionality of a healthy, adult colon and vice versa. Data of the small intestinal mucosa are not considered, because of its different physiology and interaction with gut microbes.^24^ On the basis of this analysis, we argue that a better understanding of the specific interactions between mucosal microbes and their environment is essential for exploring the full potential of gut microbes to modulate human health.

The major environmental factor shaping the mucosal biofilm is mucus. Colon mucus—in contrast to the unattached and discontinuous mucous layer in the small intestine—is present as a thick and continuous layer.^25,26^ It forms a viscoelastic, permeable gel that lubricates and protects the colon epithelial cells against foreign particles and microbial invasion while selectively allowing for the transport of gases, ions, nutrients and proteins.^27^ These selective barrier properties are determined by its biochemical composition, with the most important constituents being gel-forming mucins. Mucins are densely glycosylated glycoproteins that are cross-linked and assembled via disulphide bonds, and often negatively charged with sialic acids or sulphate groups. In the colon, Muc2 mucins are the only gel-forming mucins.^28^ Colon Muc2 mucins form two distinct layers: a firm, cell-adherent inner mucous layer, and a loose, unattached outer mucous layer.^26,29^ The inner mucous layer is a thin, lamellar, highly organised layer of densely stacked Muc2 mucins that remain anchored to the epithelial cells. It has a thickness of several hundred micrometres.^30^ The outer mucous layer is formed from the inner layer by proteolytic cleavages allowing the Muc2 mucin to expand three to four times into a loose polymeric network removable by suction.^31^ The rigidity and viscosity of mucus may further fluctuate, as mucin glycosylation patterns, their charge and their degree of cross-linking are highly responsive to the exact concentration of other mucous constituents such as water, lipids, ions, DNA, proteins, cells and cellular debris.^27^ For example, high concentrations of calcium can entirely collapse the mucus gel by facilitating protein cross-links between mucin monomers,^32^ while high acidity can decrease the viscosity of mucus by reducing the negative charges on sialic acids.^33^

Another intrinsic feature of the colon mucous layer is its rapid turnover time. A recent mouse study has revealed that the inner mucous layer is fully renewed within 1 h,^34^ while the gut epithelium tissue is self-renewed within 4–5 days.^35^ Hence, it is clear that mucus forms a remarkable and sensitive niche for microbial life, in which microbes are challenged to adapt to the biochemical dynamics determining mucous viscoelasticity and to persist within a continuously renewed environment.

We identify six microenvironmental conditions in the mucosal biofilm that have been evidenced to impact microbial colonisation and functionality (Figure 1). First, the increased rigidity of the inner mucous layer is shown to physically interfere with the migration of microbes. Mucosal microbes mostly colonise the looser, outer mucous layer, whereas the inner, firmer layer is reported to be largely devoid of or contain very low numbers of bacteria.^22,31^ In rats, the inner mucous layer was furthermore shown to have a distinct bacterial community composition.^22^ Indeed, differences in viscosity were found to significantly select for microbes with a different morphology.^36^ Low viscosity selects for short coccoid rods like *Bacteroides* spp., a moderately viscous environment is preferred by long curved rods like *Eubacterium rectale*, and a high viscosity immobilizes all bacterial groups.^36^ Gradients in mucous rigidity may further select for microbes that developed tools to attach to and/or migrate within the mucous layer. These tools are typically extracellular protein polymers like flagella, pili and fimbriae.^37^ Pili and fimbriae are mostly used for adherence to epithelial cells and mucus, while flagella additionally enable microbes to swim and swarm across the mucus. These structures have extensively been studied in human intestinal pathogens and are important for colonisation of the host tissue and virulence.^38^ However, they may also be important for mucous colonisation by beneficial microbes, as adherent pili and fimbriae have been identified in *L. rhamnosus* GG^39^ and *Bifidobacterium breve*.^40^ Moreover, a variety of *Lactobacillus* spp. was found to possess mucus-binding proteins that enable adhesion to a wide range of mucous ligands.^37^ Finally, it was suggested that commensal mucus-binding *Bacteroides* spp. utilise extracellular proteins that bind starch and other carbohydrates on the mucin glycoproteins.^41^

Interestingly, there is increasing evidence that gut microbes may actively participate in regulating mucous viscosity. A recent study with two colonies of genetically identical mice showed that the presence of a specific gut microbiome may determine the strength of the mucous barrier.^42^ The first mouse colony had an inner mucous layer that was impenetrable by bacteria or beads of the same size, whereas the inner mucous layer of the second colony was penetrable by bacteria and beads. The mucous phenotypes depended on the gut microbiome because they were transmissible by transfer of caecal microbiota to germ-free mice. At the biochemical level, evidence for a microbial impact on mucous viscosity and penetrability is provided by study of the human commensal *F. prausnitzii*. *F. prausnitzii* growth in the oxygenated mucosal biofilm was found to require free pools of thiols, such as cysteine and glutathione.^23^ These are redox mediators and are important for maintaining mucous fluidity while they can modulate reduction of the disulphide bonds that assemble the mucin glycoproteins into polymeric networks.^43^ Hence, *F. prausnitzii* growth and mucous rigidity rely on the same biochemical products, and might be closely connected.

Second, differences in rigidity between inner and outer mucous layer expose the mucosal microbes to gradients of fluid shear. Microbes that reside between the microvilli of the epithelial cells are exposed to a low fluid shear environment^44^ and experience much less physical perturbation when compared with the microbes in the outer, more turbulent mucous layer. Low fluid shear was shown to completely reprogramme the gene expression, physiology and stress resistance of several (opportunistic) pathogens, including *Salmonella enterica* serovar Typhimurium (for which virulence was also altered), *Escherichia coli, Pseudomonas aeruginosa* and *Staphylococcus aureus*.^44–46^ The latter two organisms also showed alterations in biofilm formation.^46,47^ In the case of *S. aureus*, low fluid shear conditions that altered biofilm architecture made the bacterium more resistant to antibiotic stress, lowered its resistance to oxidative stress and decreased carotenoid production.^46^ Although the impact of low fluid shear on commensal gut microbes has not been described so far, it seems plausible that mucous fluidity and shear are important determinants of mucosal biofilm structure and functionality.

Third, microbial life in the mucosal biofilm experiences gradients in oxygen, which leaks from the epithelial cells and dilutes into the mucous layer. Albenberg *et al.* recently measured a steep and radial oxygen gradient in the mice caecum going from 40 mm Hg in the intestinal tissue to deeply anaerobic conditions (<1 mm Hg) in the bulk of the lumen.^48^ For the first time, these authors demonstrated the potential of oxygen to shape the (mucosal) gut microbiome. They increased the oxygenation of mouse intestinal tissues by hyperbaric oxygen therapy, and found shifts in their faecal microbial composition. Moreover, in humans, the authors found that rectal mucosal biopsies and swabs contained a higher relative abundance of oxygen-tolerant *Proteobacteria* and *Actinobacteria* when compared with paired stool samples.

Additional evidence for oxygen gradients to shape mucosal biofilm formation is provided by the fact that prominent strictly anaerobic mucosal microbes seem to benefit from low oxygen concentrations. As mentioned previously in this text, *F. prausnitzii* species were found to tolerate low concentrations of oxygen by using an extracellular electron shuttle of flavins and thiols.^23^ This way of electron shuttling to oxygen results in a competitive growth advantage and may explain why *F. prausnitzii* is found *in vivo* and *in vitro* in the outer mucous layer and lumen/mucus interphase.^19,49,50^ Another mechanism by which gut microbes may benefit from low oxygen levels near the gut mucosa was found in *Bacteroides fragilis*. This species encodes cytochrome *bd* oxidase, an essential enzyme for oxygen consumption that was shown to stimulate its growth in the presence of nanomolar concentrations of oxygen.^51^ This feature explains why strictly anaerobic *Bacteroides* spp. are able to colonise the mouse intestine without pre-colonisation by facultative anaerobes that reduce oxygen creating a strictly anaerobic environment.^52^ Cytochrome oxidases seem to be widespread in the genome of (gut) bacterial species belonging to different phyla, among which are found many human intestinal pathogens.^51,53^ Hence, it is likely that a variety of microbes and pathogens are benefiting from low oxygen levels near the colon epithelial surface.^53^

Finally, increased oxygen availability upon migration to the colonic mucosal surface was found to enhance the adhesive and/or invasive properties of human enteric pathogens that infect colon epithelial cells, including *Salmonella enterica*,^54,55^ *Shigella flexneri*^56^ and enterohaemorrhagic *Escherichia coli*.^57^ Radial oxygen gradients in the colon mucous layer may thus also have a role in disease and the disruption of the normal microbiota.

A fourth factor shaping colon mucosal colonisation patterns is the secretion of host defense molecules such as antimicrobial peptides (AMPs) and secretory IgA, by the colon epithelial cells. In comparison with the small intestinal epithelium, colon epithelial cells are secreting much lower amounts of AMPs and sIgA than small intestinal epithelium.^25^ This is due to their different physiology: AMP-secreting Paneth cells are abundant in the small intestine but rare in the colon, and sIgA-regulation by aggregated lymphoid nodules (Peyer’s patches) in the small intestine is taken over by smaller, isolated lymphoid follicles in the colon.^25^ Yet, the antimicrobial activity in the colon mucosal biofilm should not be underestimated. It has been shown that secretions of AMPs remain trapped in the mucus and only marginally leach to the lumen.^58^ In addition, AMPs can also be produced by mouse Goblet cells, which are mucus-secreting enterocytes that are highly abundant in the colon.^59^ Although the presence of these antimicrobial components in the mucous layer impacts the colonisation ability of several microorganisms, mucosal microbes themselves are capable to regulate their secretion and thereby modify the mucosal microbiome. *Bacteroides thetaiotaomicron* has been described to shape innate immunity by modulating antimicrobial angiogenin4 production by mouse Paneth cells.^60^ Another example is the inducation of RegIIIγ production in the mouse colon, not ileum, by the mucinolytic *Akkermansia muciniphila*. As RegIIIγ exerts direct antimicrobial activity against gram-positive microbes in the intestine,^61^ *A. muciniphila* is thus able to favour its own colonisation and growth. Hence, we infer that colon mucosal biofilm formation is shaped by the build-up of host defense molecules in the thick mucous layer, with antimicrobial activities comparable to (or even greater than) those in the small intestine.

A fifth factor that determines whether microbes are able to persist within the mucous layer is the presence of a rich but challenging nutrient platform. Mucosal microbes may feed or cross-feed on ‘easy’ substrates that are leaching from the lumen in the (outer) mucous layer or that are produced in the biofilm. However, it appears that the colon mucosal nutrient platform is dominated by less degradable host-derived secretions. Indeed, the composition of the mucosal biofilm was evidenced to be enriched in asaccharolytic microbes that primarily metabolise peptones and amino acids, which are probably derived from the proteinaceous substrates from mucus and shedded epithelium.^48^ In the mouse caecum, stable isotope probing with threonine revealed that two bacterial species, *A. muciniphila* and *Bacteroides acidifaciens*, are key host-protein foragers *in vivo*.^62^ *A. muciniphila* and *Bacteroides* spp. are dominant members of the human colon microbiome^8,63^ that have been shown to degrade mucin glycoproteins.^64,65^ *Bacteroides* spp. and *A. muciniphila* have evolved rather different strategies to forage on mucin. Although *Bacteroides* spp. have broad polysaccharide degradation potential^66^ and only turn to mucin glycans when dietary glycans are depleted,^67^ *A. muciniphila* has specialized to utilise mucins as a sole carbon and nitrogen source.^63,64^ For *B. thetaiotaomicron*, foraging on mucin glycans was found to be important for successful colonisation of the mouse gut, because deletion of the involved gene cassettes was shown to compromise its transmission from gnotobiotic mother to offspring.^68^ Hence, its capacity to thrive on mucin glycans and live in close proximity to the intestinal epithelial surface seems to be an evolutionary trait allowing for its stable and continuous colonisation of the gut. For *A. muciniphila*, foraging on mucin seems not to confine the species to the mucosal biofilm niche. In our dynamic *in vitro* model of the proximal and distal colon microbiome (SHIME),^69,70^ *A. muciniphila* was found to abundantly colonise the distal colon compartment in the absence of a mucosal biofilm. Moreover, supplementation of mucin to this model resulted in a strong and quick increase in luminal *A. muciniphila* qPCR counts (4.5 log increase in 2 days) (unpublished data). In addition, *A. muciniphila* seems not to bind to human colon mucus.^71^ Considering *A. muciniphila*’s unique specialisation to grow on mucin, we infer that its *in vivo* niche is associated with mucus regardless of their embedment within the colon mucosal biofilm or not.

Although some mucosal species directly benefit from host-secreted mucin, their activity was found to depend on the presence of other community members.^62,72^ Indeed, complete degradation of the heterogeneous and complex structure of mucin glycoproteins requires a diverse set of enzymes,^73^ and the *in vitro* mucin degradation potential of microbes is phylogenetically widespread.^74,75^ On the other hand, specialist mucin degraders may stimulate the growth of other mucosal microbes. For example, *Bacteroides* spp. are known to release sulphate from mucin glycans, which makes them more accessible for further degradation by other microbes^76^ and may stimulate the growth of sulphate-reducing bacteria.^77^

Finally, microbes may enhance their persistence in the colon mucosal biofilm by specializing to colonise niches at the colon epithelial surface. An example of such a niche is the intestinal crypt. Crypts are vulnerable sites of the gut epithelium as they contain stem cells and are the site of epithelial proliferation and restitution. In healthy rodents, crypt-associated microbes have been detected in the caecum and proximal colon, but not in the distal colon and rectum.^36,78,79^ Likewise, healthy human rectal crypts were found to be devoid of bacteria.^80,81^ Crypt-associated microbes in the human proximal colon have not been studied so far owing to the difficulties with obtaining correct and unbiased samples. The crypt micro-environment is proposed to be characterized by a low mucous viscosity,^36^ a high partial oxygen pressure that selects for aerobic species in mice^79^ and the presence of specific types and concentrations of host glycans mediating saturable crypt occupancy by *Bacteroides* spp.^17^ Interestingly, glycan-mediated crypt occupancy by *Bacteroides* was found to be responsible for its stable colonisation of the gut, even in the event of enteric infection or following antibiotic treatment.^17^ The authors therefore proposed that crypt-associated microbiota might represent bacterial reservoirs of founder cells that repopulate the gut following disruption by infections or antibiotic consumption.^17^

Altogether, it has become evident that the colon mucosa is a unique and highly structured gut environment. The combination of radial gradients in rigidity, fluid shear and oxygen concentrations, and the presence of antimicrobial host secretions and a distinct nutrient pool creates a variety of microbial microenvironments that result in a specific biofilm stratification or architecture that impact mucosal biofilm composition and functionality. On the other hand, microbes seem to actively participate in shaping the mucosal environment, for example, by degrading mucus and affecting mucous viscosity or triggering the production of AMPs.

Although the composition of the mucosal biofilm seems to correlate with health status, insight into the intrinsic relation between mucosal microbes and their environment could hold exciting perspectives for monitoring and modulating human health. Particular interest thereby goes to the interrelated and simultaneous impact of the different microenvironmental factors on mucosal biofilm functioning, in both health and disease. Overall, such research efforts should help in identifying (i) what mucosal species and/or microbial functionalities are crucial for maintaining a healthy mucosal barrier, (ii) how the immune system copes with these mucosal microbes and what role mucolytic activity from specific gut microbes has in modulating human health.

## Figures and Tables

**Figure 1:**
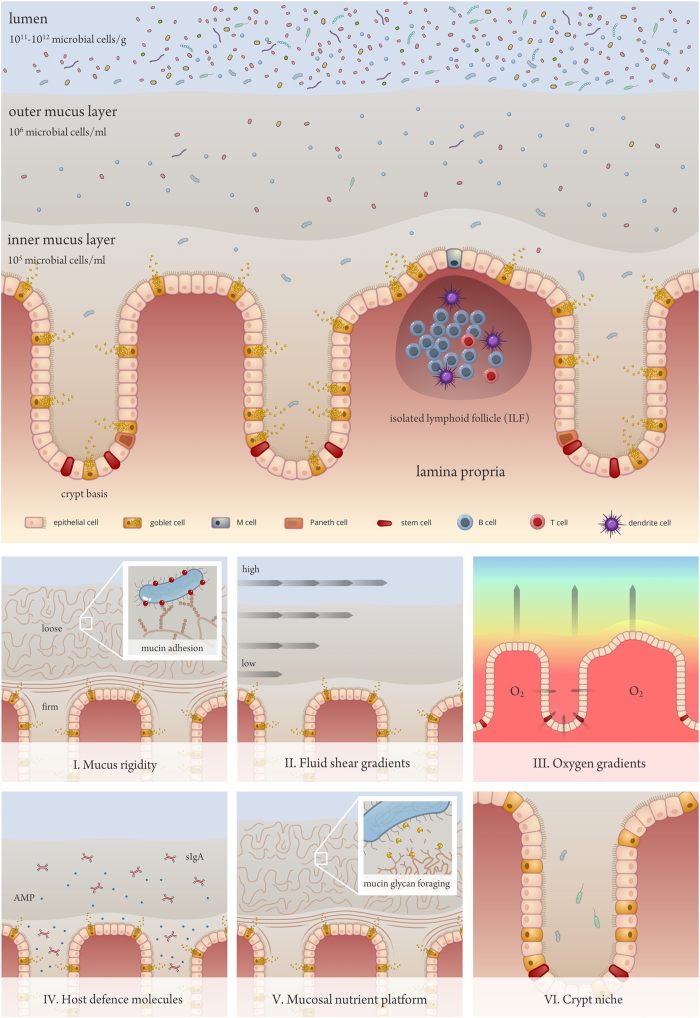
Schematic overview of six microenvironmental factors near the colon epithelium that are evidenced to impact mucosal microbial colonisation and functionality.
